# MiR-129-5p regulates cell proliferation and apoptosis via IGF-1R/Src/ERK/Egr-1 pathway in RA-fibroblast-like synoviocytes

**DOI:** 10.1042/BSR20192009

**Published:** 2019-11-28

**Authors:** Yan Zhang, Ni Yan, Xiaoqing Wang, Yanhai Chang, Yu Wang

**Affiliations:** 1 Department of Endocrinology, Shaanxi Provincial People’s Hospital, Xi’an 710068, China; 2 Outpatient Office, Shaanxi Provincial People’s Hospital, Xi’an 710068, China; 3 Department of Orthopaedics, Shaanxi Provincial People’s Hospital, Xi’an 710068, China; 4 Department of Orthopaedics, Xi’an Central Hospital, Xi’an 710003, China

**Keywords:** IGF-1R, MiR-129-5p, Rheumatoid arthritis, Src/ERK/Egr-1 pathway

## Abstract

It is reported that miR-129-5p plays an important role in various diseases, but its effect on rheumatoid arthritis (RA) and the potential mechanism remain to be clarified. In the present research, we aimed to investigate the effect of miR-129-5p on RA and the special molecular mechanism. First, the expression of miR-129-5p was analyzed in RA patients and RA Fibroblast-like synoviocytes (RA-FLSs) by RT-PCR assay. The cell viability, apoptotic rate and the relative expression of caspase-3 and caspase-8 were measured by CCK-8, Annexin-FITC/propidium iodide (PI) and ELISA, respectively. Luciferase reporter assay was performed to investigate the target of miR-129-5p. The results revealed that the expression of miR-129-5p was down-regulated in RA patients and RA-FLSs. In addition, miR-129-5p inhibited cell proliferation and induced apoptosis of RA-FLS. Furthermore, luciferase reporter assay demonstrated that insulin-like growth factor-1 receptor (IGF-1R) was the direct target of miR-129-5p, and IGF-1R promoted cell proliferation and inhibited apoptosis by activating Src/ERK/Egr-1 signaling. Furthermoremore, the Src/ERK/Egr-1 signaling pathway was suppressed by miR-129-5p. Collectively, the results of the present study suggested that miR-129-5p regulated cell proliferation and apoptosis via IGF-1R/Src/ERK/Egr-1 signaling pathway in RA.

## Introduction

Rheumatoid arthritis (RA) is a joint disease caused by chronic inflammation, and the most important clinical feature is chronic arthritis, which will eventually lead to bone destruction, cartilage, bone damage and disability [1,2]. RA mostly occurs in women aged 40–60, the incidence of whom is two to three times more than men [3]. At present, the main clinical treatment strategy of RA is drug therapy, including immunosuppressive drugs and biological agents. However, these therapies induce a general drug resistance that increases the risk of infectious diseases and cancer [4,5]. Therefore, it is urgent to study new therapeutic approaches to treat RA.

MicroRNAs (miRNAs) are short non-coding RNA, which regulate the transcription of target genes through the specific binding of 3′untranslated region (UTR) to target genes [6]. A large number of studies have shown that most miRNAs in various cancer cells are abnormally expressed. Therefore, miRNAs could be used as regulators of oncogenes, anti-oncogenes or metastasis genes [7,8]. In recent years, increasing evidences have shown that miRNAs play important roles in the occurrence and development of RA by regulating cell viability, apoptosis, and invasion [9,10]. MiR-129-5p is transcribed from two genes, *miR-129-1* and *miR-129-2*, which has been reported to be involved in the occurrence and development of various diseases [11]. Previous studies have shown that miR-129-5p is an important tumor suppressor, and overexpression of miR-129-5p significantly reduces the proliferation, migration and invasion of cancer cells [12]. However, the role of miR-129-5p in the proliferation and migration of RA cells remains unclear.

Insulin-like growth factor-1 (IGF-1), which has high homology with insulin, plays a crucial role in brain development, nerve cell growth, cell proliferation and differentiation by activating multiple signaling pathways through binding to the receptor (IGF-1R) [13,14]. Clinically, patients with RA have a high level of IGF-1/IGF-1R and a high incidence of malignant diseases [15]. IGF-1 binds to IGF-1R and then activates several signal transduction pathways and kinases in various cells, such as extracellular signal-regulated kinase-1/2 (ERK-1/2) and Src/MAPK signal transduction pathways. [16]. Meghan et al. reported that IGF-1R acts as a stimulator to activate its downstream Src/MAPK/Egr-1 signaling pathway, which regulates secretory clusterin protein (sCLU) pro-survival pathway and plays an important role in radiation resistance in cancer therapy [17]. The Src/ERK/Egr-1 signaling pathway is the most typical downstream pathway of IGF-1/IGF-1R, which participates in various physiological processes of cells as well as the occurrence and development of various cancers.

In the present study, we explored the effect of miR-129-5p on RA and its potential mechanisms. The results of the present study suggested that miR-129-5p inhibits cell proliferation and induces apoptosis of RA-FLS via Src/ERK/Egr-1 signaling by directly target IGF-1R in RA.

## Materials and methods

### Patients

From August 2017 to October 2018, 15 patients with RA, including 10 males and 5 females, aged 50–70 years (average age 62 years), were treated in hospital. Pathological examination confirmed the diagnosis. In addition, 2 ml peripheral blood samples were collected from all these patients, and peripheral blood samples from 12 healthy volunteers were taken as controls. NC: normal health control serum (*n*=12); RA: RA serum (*n*=15); NC-synovial: normal health control synovial tissue (*n*=12); RA-synovial: RA synovial tissue (*n*=15); NC-FLSs: normal human Fibroblast-like synoviocytes; RA-FLSs: RA Fibroblast-like synoviocytes. The study was approved by the local Ethics Committee and the written consent of all selected patients was obtained.

### Cell lines

The normal human FLSs and rheumatoid arthritis (RA) FLSs were purchased from Cell Applications (San Diego, CA, U.S.A.). The cells were cultured in DMEM containing 10% FBS and stored in a humidified incubator with 5% CO_2_ at 37°C.

### Reagents

Primescript™ reverse transcription kit and SYBR® Premix ExTaq™ II were purchased from Genetime Biotechnology Co., Ltd. (Shanghai, China). DMEM and 10% FBS were purchased from Thermo Fisher Scientific Biotech (Massachusetts, U.S.A.). Antibodies including anti-IGF-1R, anti-p-src, anti-p-ERK, anti-ERK, anti-Egr-1 and anti-c-src were all obtained from Invitrogen Biotechnology Co., Ltd. (Massachusetts, U.S.A.). GAPDH was purchased from Abcam Inc. (Cambridge, U.K.). ELISA kits were purchased from MSKBIO Technology Ltd. (Wuhan, China).

### Transfection

MiR-129-5p mimics, pcDNA3.1-IGF-1R, Egr-1-siRNA, miR-129-5p inhibitor, miR-NC and the vector pcDNA3.1 were designed and synthesized by Tsingke Biotech Co., Ltd. (Beijing, China). The plasmid vectors were transfected into RA-FLAs or NC-FLSs using Lipofectamine 2000 (Invitrogen, U.S.A.) according to the manufacturer’s instructions. The cells were incubated for 24 h before using in subsequent experiments.

### ELISA

According to the manufacturer’s instructions, the levels of caspase-3 and caspase-8 were measured by ELISA kit. The absorbance at 450 nm was measured with a microplate reader.

### Cell apoptosis analysis

The RA-FLAs or NC-FLSs were plated on six-well plates and transfected with miR-129-5p mimics or miR-129-5p inhibitor, and cells were incubated for 24 h. Finally, the cells were collected and incubated with Annexin-V and propidium iodide (PI) for 40 min. The incidence of apoptosis was evaluated by the flow cytometer (FCM).

### Cell viability

First, 200 μl cell suspension was inoculated into 96-well plate with 1 × 10^4^ cells per well, and 1.5 ml lovastatin solution was added in the lower layer. The cell suspension was cultured at 37°C for 8 h. Then CCK-8 and 4% polyformaldehyde were added to each well for 20 min and Crystal Violet staining was added for 15 min. After drying, the cells migrating to the surface under microscopy were counted. The above experiments were repeated three times.

### Luciferase reporter assay

The wild-type (WT IGF-1R) or mutant (MUT IGF-1R) IGF-1R 3′-UTR sequences in the predicted target were amplified by PCR and linked to the pcDNA3.1 vectors (Tiangen, Beijing). Then, 293T cells were transfected with miR-129-5p (50 nM) or miR-NC (50 nM). After 24 h of transfection, luciferase activity was measured by the Dual-luciferase activity assay system following the manufacturer’s instructions.

### RT-qPCR

The total RNA was extracted from cells using TRIzol reagent (Invitrogen, Carlsbad, CA, U.S.A.) following to the guidelines. A total of 1 μg RNA was used for the preparation of cDNA using the Revert Aid™ First Strand cDNA Synthesis kit (TaKaRa, Shiga, Japan) following the manufacturer’s guidelines. The RT-PCR cycling conditions consisted of: 95°C for 10 min; then 32 cycle amplification for 30 s at 95°C, 20 s at 55°C, 20 s at 72°C; followed by 1 min at 72°C. The level of mRNA was normalized to β-actin expression using the 2^−ΔΔ*C*_t_^ method.

### Western blot

Cells were harvested and centrifuged at 8000 rpm for 60 min. The lysates were separated on SDS/polyacrylamide gel (12%) and transferred on to a PVDF membranes. Afterward, the membranes were blocked with fresh PBS with 5% skimmed milk at room temperature overnight and incubated with primary antibody for 1 h at 4°C. Densitometric measurements were performed using ImageJ computer software.

### Statistical analyses

All statistical analysis were performed using SPSS 22.0 (Chicago, IL, U.S.A.). The data are presented as the mean ± SEM. The differences between groups were analyzed by Student’s *t* test, and ANOVA was used to compare the differences between multiple groups. When *P*<0.05, the difference was considered to be statistically significant.

## Results

### miR-129-5p expression is down-regulated in RA patients and RA-FLSs

**Figure 1:**
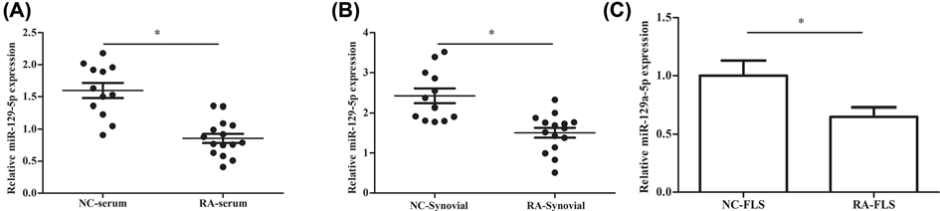
miR-129-5p expression is down-regulated in RA patients and FLSs NC: normal health control serum (*n*=12); RA: rheumatoid arthritis serum (*n*=15); NC-synovial: normal health control synovial tissue (*n*=12); RA-synovial: rheumatoid arthritis synovial tissue (*n*=15); RA-FLSs: rheumatoid arthritis fibroblast-like synoviocytes. (**A**) The relative expression of miR-129-5p in NC-serum and RA-serum group. (**B**) The relative expression of miR-129-5p in NC-synovial and RA-synovial group. (**C**) The relative expression of miR-129-5p in NC-FLS and RA-FLS groups. The experiments were repeated three times and data are presented as the mean ± SEM. ‘*’ means compared with NC-serum, NC-synovial or NC-FLS group; *P*<0.05.

In order to determine the expression of miR-129-5p in RA patients and RA-FLSs, 15 tissues and serums with RA as well as 12 normal human control tissues and serums were analyzed by qRT-PCR. The results showed that the relative expression of miR-129-5p was signifcantly decreased in RA-serum compared with the NC-serum (Figure 1A). Similarly, the expression of miR-129-5p in RA synovial tissue was down-regulated significantly compared with normal human control synovial tissue (Figure 1B). Furthermore, the relative expression of miR-129-5p also showed the same trend in the RA-FLSs (Figure 1C).

### miR-129-5p inhibits cell proliferation and induces apoptosis of RA-FLS

**Figure 2:**
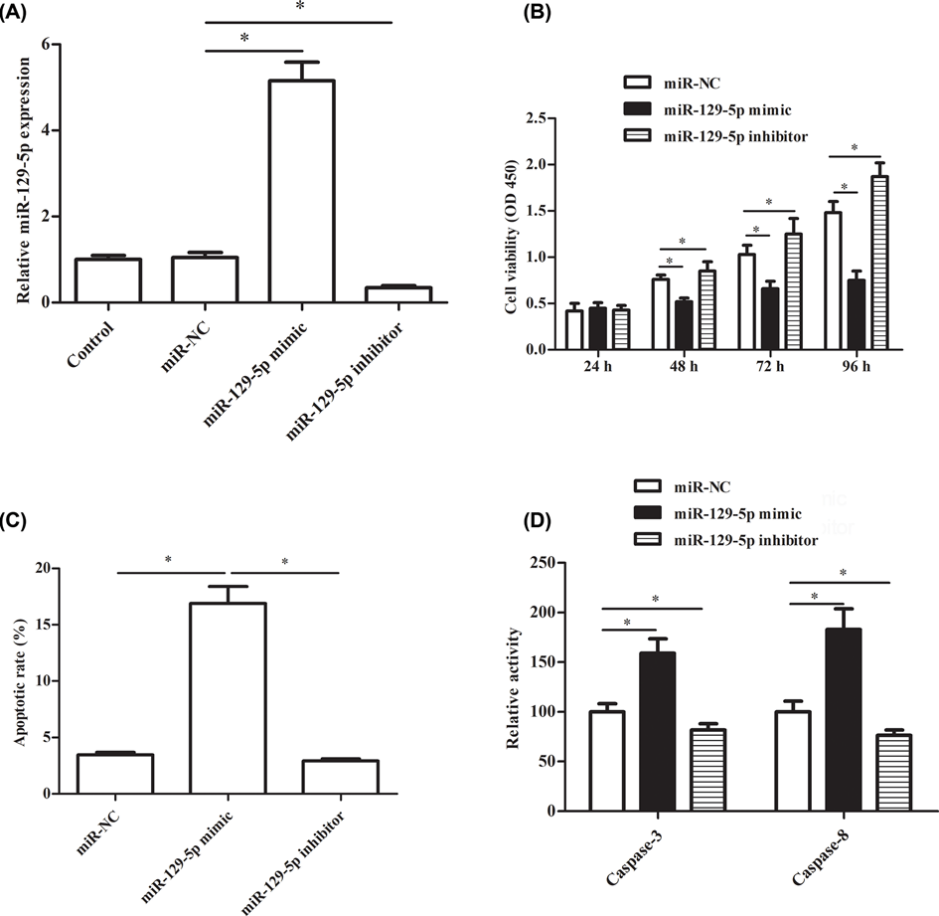
miR-129-5p inhibits cell proliferation and induces apoptosis of RA-FLS RA-FLSs were transfected with the miR-129-5p mimics (miR-129-5p group), miR-129-5p inhibitors (0.2 μg, miR-129-5p inhibitors group) or corresponding controls (miR-NC group), respectively. (**A**) The relative expression of miR-129-5p after transfection with miR-NC, miR-129-5p mimic or miR-129-5p inhibitor. (**B**) Cell viability after transfection with miR-NC, miR-129-5p mimic or miR-129-5p inhibitor. (**C**) Apoptotic rate (%) after transfection with miR-NC, miR-129-5p mimic or miR-129-5p inhibitor. (**D**) The relative activity of caspase-3 and caspase-8 after transfection with miR-NC, miR-129-5p mimic or miR-129-5p inhibitor. The experiments were repeated three times and data are presented as the mean ± SEM. ‘*’ means compared with miR-NC group; *P*<0.05.

To determine the role of miR-129-5p in RA, RA-FLSs were transfected with miR-129-5p mimics or inhibitors to alter the expression of miR-129-5p. As expected, the expression of miR-129-5p in miR-129-5p mimics group was significantly increased than the miR-NC group. In addition, after miR-129-5p inhibitor treatment, the expression of miR-129-5p was significantly decreased compared with the miR-NC group (Figure 2A). Besides, the cell viability was decreased significantly in the miR-129-5p mimic group than the miR-NC group after 48, 72 and 96 h, and the decreased trend became more and more obvious (Figure 2B). While miR-129-5p inhibitor transfection showed a contrary effect on cell viability (Figure 2B). In addition, treatment with the apoptotic rate (%) was up-regulated significantly in miR-129-5p mimic transfection group and down-regulated significantly in the miR-129-5p inhibitors transfection group compared with the miR-NC group (Figure 2C). Furthermore, the relative activity of caspase-3 and caspase-8 were increased after treatment with miR-129-5p mimic and decreased by miR-129-5p inhibitor transfection compared with treatment with miR-NC (Figure 2D).

### IGF-1R is the direct target of miR-129-5p

**Figure 3:**
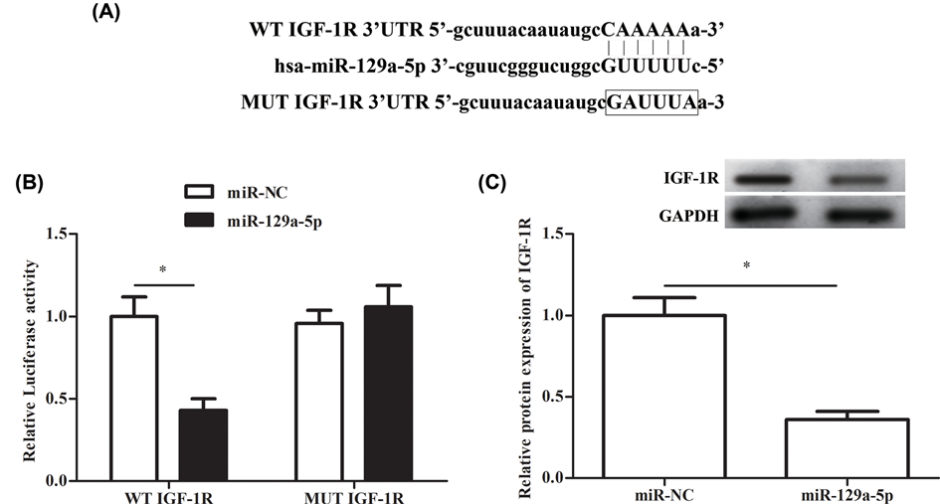
Identification of IGF-1R as a direct target of miR-129-5p RA-FLSs were transfected with the miR-129-5p mimics (miR-129-5p group) or corresponding controls (miR-NC group), respectively. (**A**) Prediction of miR-129-5p potential binding sites, and mutations on IGF-1R 3′UTR. (**B**) The relative luciferase activity in miR-129-5p and miR-NC group. (**C**) The relative protein expression of IGF-1R in miR-129-5p and miR-NC control group. ‘*’ means compared with miR-NC group; *P*<0.05. The experiments were repeated three times and data are presented as the mean ± SEM. GAPDH was used as an invariant internal control for calculating protein-fold changes.

We used TargetScan to predict the target of miR-129-5p. As shown in Figure 3A, the 3′UTR of IGF-1R specifically bound to miR-129-5p, which may be the target gene of miR-129-5p (Figure 3A). To verify this speculation, Luciferase reporter assay was performed, and the result showed that the relative luciferase activity was significantly reduced by co-transfection with miR-129-5p mimic and WT-IGF-1R 3′UTR, while treatment with miR-129-5p mimic and MUT-IGF-1R 3′UTR showed no significant difference (Figure 3B). Furthermore, the relative protein expression of IGF-1R was significantly decreased after treatment with miR-129-5p mimic compared with the miR-NC group in RA-FLSs (Figure 3C). These results showed that IGF-1R was a direct target of miR-129-5p, and miR-129-5p inhibited the expression of IGF-1R.

### miR-129-5p regulates RA-FLS proliferation and apoptosis through targeting IGF-1R

**Figure 4:**
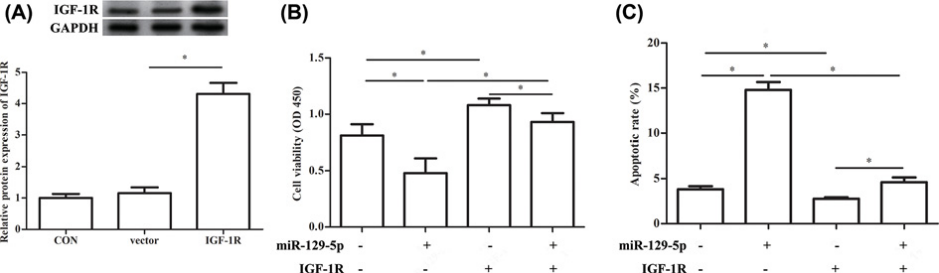
miR-129-5p regulates RA-FLS proliferation and apoptosis through targeting IGF-1R RA-FLSs were transfected with the miR-129-5p mimics (miR-129-5p group), pcDNA3.1-IGF-1R plasmid (IGF-1R group), corresponding controls (miR-NC group) or pcDNA3.1 vector (vector group). (**A**) The relative protein expression of IGF-1R in control group, pcDNA3.1 vector group and pcDNA3.1-IGF-1R group. (**B**) The cell viability after transfection with miR-129-5p, pcDNA3.1-IGF-1R or both. (**C**) Apoptotic rate (%) after transfection with miR-129-5p, pcDNA3.1-IGF-1R or both. The experiments were repeated three times and data are presented as the mean ± SEM. ‘*’ means compared with pcDNA3.1 vector or control (untreated) group. GAPDH was used as an invariant internal control for calculating protein-fold changes.

To further confirm the role of IGF-1R in RA, we constructed an overexpression vector of IGF-1R. The results showed that the protein expression of IGF-1R was increased significantly after transfection with pcDNA3.1-IGF-1R compared with treatment with pcDNA3.1 vector (Figure 4A). The cell viability was decreased significantly in the miR-129-5p mimic group than the untreated group, while pcDNA3.1-IGF-1R significantly increased the cell viability. However, co-transfection with miR-129-5p mimic and pcDNA3.1-IGF-1R significantly up-regulated the cell viability compared with the miR-129-5p mimic group (Figure 4B). Besides the apoptotic rate was increased significantly by miR-129-5p mimic transfection, while treatment with pcDNA3.1-IGF-1R inhibited the apoptotic rate significantly. In addition, co-transfection with miR-129-5p mimic and pcDNA3.1-IGF-1R significantly down-regulated the apoptotic rate compared with miR-129-5p mimic group (Figure 4C).

### IGF-1R promotes cell proliferation and inhibits apoptosis by activating Src/ERK/Egr-1 signaling

**Figure 5:**
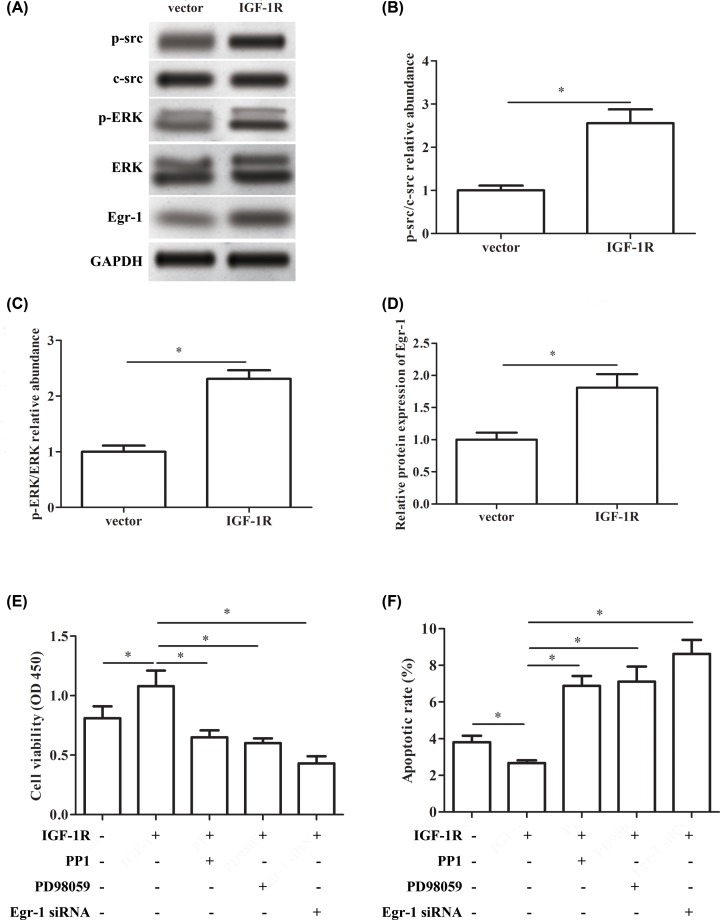
IGF-1R promotes cell proliferation and inhibits apoptosis by activating Src/ERK/Egr-1 signaling RA-FLSs were transfected with pcDNA3.1 vector (vector group), pcDNA3.1-IGF-1R vector (IGF-1R group) or Egr-1-siRNA (Egr-1-siRNA group), or treated by the inhibitors (PP1or PD98059) of src and ERK signaling. (**A**) The relative protein expression of p-src, c-src, p-ERK, ERK and Egr-1 after transfection with pcDNA3.1 or pcDNA3.1-IGF-1R vector. (**B**) The p-src/c-src relative abundance after transfection with pcDNA3.1 or pcDNA3.1-IGF-1R vector. (**C**) The p-ERK/ERK relative abundance after transfection with pcDNA3.1 or pcDNA3.1-IGF-1R vector. (**D**) The relative protein expression of Egr-1 after transfection with pcDNA3.1 or pcDNA3.1-IGF-1R vector. (**E**) The cell viability after transfection with pcDNA3.1-IGF-1R or Egr-1-siRNA, or treated by the inhibitors (PP1or PD98059), respectively. (**F**) Apoptotic rate (%) after transfection with pcDNA3.1-IGF-1R or Egr-1-siRNA, or treated by the inhibitors (PP1or PD98059), respectively. The experiments were repeated three times and data are presented as the mean ± SEM. ‘*’ means compared with pcDNA3.1 vector, control (untreated) group or pcDNA3.1-IGF-1R group. GAPDH was used as an invariant internal control for calculating protein-fold changes.

Next, we explored the effect and mechanism of IGF-1R in the process of RA. As shown in Figure 5A, the expression of p-src, c-src, p-ERK, ERK and Egr-1 was up-regulated by pcDNA3.1-IGF-1R transfection compared with the pcDNA3.1 vector group (Figure 5A). Meanwhile, pcDNA3.1-IGF-1R transfection significantly increased the relative abundance of p-src/c-src and p-ERK/ERK (Figure 5B,C). Similarly, the protein expression of Egr-1 was increased significantly in pcDNA3.1-IGF-1R transfection group compared with the pcDNA3.1 vector group (Figure 5D). PP1 is an effective selective Src family inhibitor. PD98059 is a non-ATP competitive ERK inhibitor, which specifically inhibits ERK signaling pathway. PP1 or PD98059 treatment significantly attenuated the increased cell viability that was induced by pcDNA3.1-IGF-1R transfection. Similarly, treatment with pcDNA3.1-IGF-1R and Egr-1 siRNA significantly decreased the cell viability compared with the pcDNA3.1-IGF-1R group (Figure 5E). The results of Annexin-FITC/PI showed that the apoptotic rate was decreased significantly by pcDNA3.1-IGF-1R, while treatment with pcDNA3.1-IGF-1R and PP1 or PD98059 increased the apoptotic rate significantly compared with the pcDNA3.1-IGF-1R group. Similarly, pcDNA3.1-IGF-1R and Egr-1 siRNA treatment significantly increased the apoptotic rate compared with the pcDNA3.1-IGF-1R group (Figure 5F).

### Src/ERK/Egr-1 signaling is suppressed by miR-129-5p

**Figure 6:**
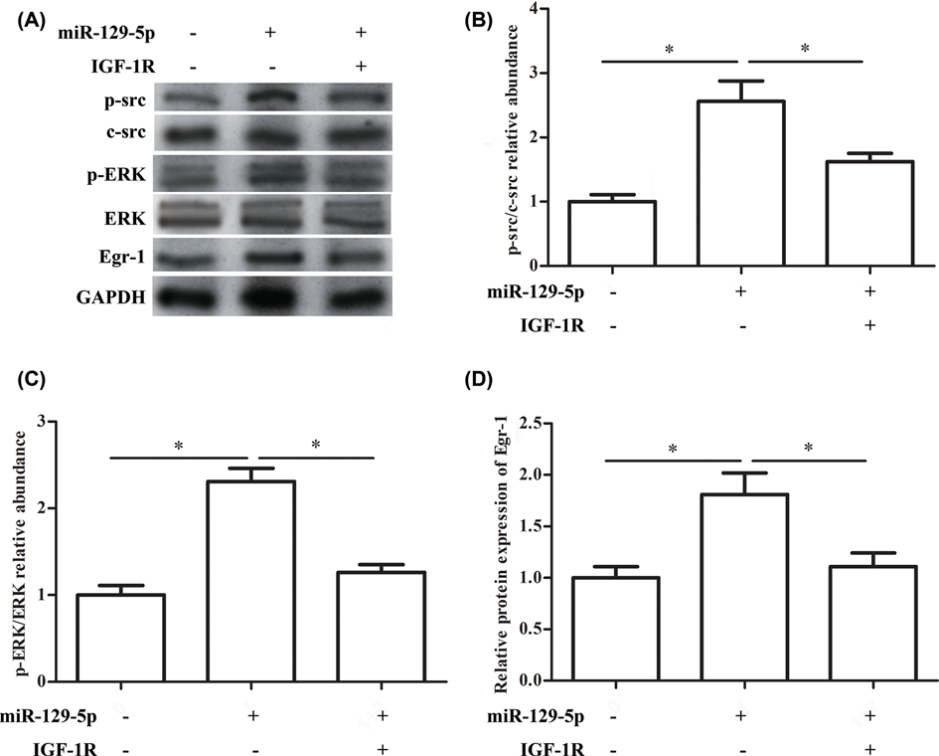
Src/ERK/Egr-1 signaling is suppressed miR-129-5p RA-FLSs were transfected with miR-129-5p mimic (miR-129-5p group), pcDNA3.1-IGF-1R (IGF-1R group) or both. (**A**) The relative protein expression of p-src, c-src, p-ERK, ERK and Egr-1 after transfection with miR-129-5p mimic, pcDNA3.1-IGF-1R or both. (**B**) The p-src/c-src relative abundance after transfection with miR-129-5p mimic, pcDNA3.1-IGF-1R or both. (**C**) The p-ERK/ERK relative abundance after transfection with miR-129-5p mimic, pcDNA3.1-IGF-1R or both. (**D**) The relative protein expression of Egr-1 after transfection with miR-129-5p mimic, pcDNA3.1-IGF-1R or both. The experiments were repeated three times and data are presented as the mean ± SEM. ‘*’ means compared with control (untreated) group or miR-129-5p (miR-129-5p mimic) group. GAPDH was used as an invariant internal control for calculating protein-fold changes.

As shown in Figure 6A, the results indicated that the protein expression of p-src, c-src, p-ERK, ERK and Egr-1 was down-regulated after treatment with miR-129-5p mimics, while the inhibited effect of miR-129-5p mimics was partly reversed by pcDNA3.1-IGF-1R (Figure 6A). In addition, when RA-FLSs were transfected with miR-129-5p mimics, the relative expression of p-src/c-src and p-ERK/ERK were significantly increased, while after further transfecting with pcDNA3.1-IGF-1R, the relative expression of p-src/c-src and p-ERK/ERK were significantly decreased (Figure 6B,C). Similarly, miR-129-5p mimics significantly increased the protein expression of Egr-1, and pcDNA3.1-IGF-1R reversed the effect (Figure 6D). These results indicate that Src/ERK/Egr-1 signaling pathway is suppressed by miR-129-5p.

## Discussion

In the present study, we focused on the effect of miR-129-5p on cell proliferation and apoptosis in human RA cell line RA-FLS as well as the special regulatory mechanism. The results showed that the expression level of miR-129-5p was significantly decreased in RA tissues and peripheral blood samples. Overexpression of miR-129-5p significantly reduced the cell viability and induced apoptosis. Further studies have found that IGF-1R, as a target of miR-129-5p, regulates Src/ERK/EGR-1 pathway and makes it as an important regulatory gene in RA. Therefore, miR-129-5p regulated RA cell proliferation and apoptosis via IGF-1R/Src/ERK/EGR-1 signaling pathway.

Studies have shown that abnormal expression of miRNAs in RA cells is involved in the pathogenesis of RA [18]. Previous research reports the expression of miR-129-5p, miR-26a, miR-125b, miR-143, miR-145 and miR-99a was significantly down-regulated in aberrant smooth muscle cells, in which miR-129-5p was the most down-regulated [19]. Our study showed similar results that miR-129-5p was down-regulated in tissue and blood samples of RA patients. Previous studies have shown that miR-129-5p plays an important role in various pathological processes, including inflammation [20]. Diao et al. [21] reported that miR-129-5p induced U87 and U251 cell apoptosis and blocked the cell cycle. Furthermore, when gastric cancer cells were transfected with miR-129-5p mimics, the cell viability, colony formation ability, migration and invasion were significantly decreased, whereas miR-129-5p inhibitors transfection showed opposite results [22]. In addition, study has shown that overexpression of miR-129-5p significantly inhibits cell proliferation, migration and promotes apoptosis in chondrosarcoma [23]. Our results showed that miR-129-5p inhibited cell proliferation and induced cell apoptosis, inhibited the development of RA, which was consistent with previous research findings. These results indicate that miR-129-5p plays an important regulatory role in many human diseases, and participates in the regulation of multiple signaling pathways. Therefore, targeting miR-129-5p may be a potential therapy for some human diseases.

Our studies have shown that miR-129-5p binds specifically to IGF-1R 3′UTR, that is, IGF-1R is the target of miR-129-5p. As a receptor of IGF-1, IGF-1R exerts various biological functions by activating different signaling pathways in various diseases. Yang et al. [24] reported that IGF-1/IGF-1R inhibited the apoptosis of retinal neurons hypoxia-induced via triggering ERK-1/2 and Akt signaling pathways. In addition, increasing reports showed that IGF-1/IGF-1R plays an important role in the development of RA. Marotte et al. [25] found that IGF-1R promotes TNF-α-induced inflammation by activating ERK-1/2 pathway in RA synovial fibroblasts. It has been reported that IGF-1R is highly expressed in RA T cells, and it stimulates the production of IL-10 by binding with IGF-1, activates RA T cells and promotes the progress of RA [18]. Furthermore, previous study has shown that the IGF-1R, ERK and Akt pathways are dephosphorylated by NVP-AEW541 to promote angiogenesis, inhibit osteoclast formation and further alleviate RA symptoms [26]. In this study, we found that activating the signal of Src/ERK/Egr-1 promoted the proliferation of FA-FLSs, inhibited the apoptosis and reversed the inhibition of miR-129-5p.

The IGF-1R/Src/Egr-1 pathway is an important survival system under many biological and pathological conditions [17]. Meghan et al. [17] reported that the IGF-1R/Src/ERK/Egr-1 signaling pathway is activated by radiation stress, and regulates the secretory clusterin protein cascade pathway, which is important for radiation resistance in cancer treatment. Furthermore, ERK-1/2 pathway is activated by IL-18bPa, which decreases the biological activity of IL-18 induced by TNF-α in rheumatoid arthritis synovial fibroblasts. Thus, ERK1/2 signaling pathway plays an important role in the occurrence and development of RA [25]. Similarly, our results showed that Src/ERK/Egr-1 signaling was activated by IGF-1R to promote cell proliferation and inhibited apoptosis.

In summary, our current study reveals that miR-129-5p regulated cell proliferation and apoptosis via IGF-1R/Src/ERK/Egr-1 pathway in RA. It suggested that miR-129-5p may be a new proliferation and apoptosis related gene in RA. In the future, the search for a deep and reasonable mechanism for the role of miR-129-5p will help us to understand its function more comprehensively, and finally find a new method for the treatment of human diseases. Furthermore, understanding the dysregulated of Src/ERK/Egr-1 pathway expressed by miR-129-5p/IGF-1R in RA is a great significance to improve our understanding of the biological basis of RA development and progress and the potential of RA treatment.

## Abbreviations

CCK-8: Cell Counting Kit-8
ERK-1/2: extracellular signal-regulated kinase-1/2
GAPDH: glyceraldehyde-3-phosphate dehydrogenase
IGF-1: insulin-like growth factor-1
IGF-1R: insulin-like growth factor-1 receptor
IL-18: interleukin 18
miRNA: microRNA
NC-FLS: normal human Fibroblast-like synoviocyte
PI: propidium iodide
PP1: Scr family inhibitor
RA: rheumatoid arthritis
RA-FLS: RA Fibroblast-like synoviocyte
UTR: untranslated region
